# A randomized controlled non-inferiority trial of primary care-based facilitated access to an alcohol reduction website (EFAR-FVG): preliminary results

**DOI:** 10.1186/1940-0640-10-S2-O29

**Published:** 2015-09-24

**Authors:** Pierluigi Struzzo, Roberto Della Vedova, Donatella Ferrante, Nicholas Freemantle, Charilaos Lygidakis, Francesco Marcatto, Emanuele Scafato, Francesca Scafuri, Costanza Tersar, Paul Wallace

**Affiliations:** 1 Research & innovation Area, Regional Centre for the Training in Primary Care, Monfalcone, Italy; 2 University of Trieste, Department of Life Sciences, Italy; 3 Department of Primary Care and Population Health, University College London, London, UK; 4 Movimento Giotto, Bologna, Italy; 5 Istituto Superiore di Sanità, WHO Collaborating Centre for Research and Health Promotion on Alcohol and Alcohol-Related Health Problems, Osservatorio Nazionale Alcol, Centro Nazionale di Epidemiologia, Sorveglianza e Promozione della Salute, Rome, Italy; 6 National Institute of Health Research Clinical Research Networks, University of Leeds, Leeds, UK

## Background

The effectiveness of brief interventions for risky drinkers by GPs is well documented.[1] However, implementation levels remain low. Facilitated access to an alcohol reduction website offers an alternative to standard face-to-face intervention, but it is unclear whether it is as effective.[2] This study evaluates whether online brief intervention, through GP facilitated access to an alcohol reduction website for risky drinkers, is not inferior to the face-to-face brief intervention conducted by GPs.

## Material and methods

In a northern Italy region participating GPs actively encouraged all patients age 18 attending their practice, to access an online screening website based on AUDIT-C.[3] Those screening positive underwent a baseline assessment with the AUDIT-10[4] and EQ-5D[5] questionnaires and subsequently, were randomly assigned to receive either online counselling on the alcohol reduction website (intervention) or face-to-face intervention based on the brief motivational interview[6] by their GP (control). Follow-up took place at 3 and 12 months and the outcome was calculated on the basis of the proportion of risky drinkers in each group according to the AUDIT-10.

## Results

More than 50% (n= 3974) of the patients who received facilitated access logged-on to the website and completed the AUDIT-C. Just under 20% (n = 718) screened positive and 94% (n= 674) of them completed the baseline questionnaires and were randomized. Of the 310 patients randomized to the experimental Internet intervention, 90% (n = 278) logged-on to the site. Of the 364 patients of the control group, 72% (263) were seen by their GP. A follow-up rate of 94% was achieved at 3 months.

## Conclusions

The offer of GP facilitated access to an alcohol reduction website appears to be an effective way of identifying risky drinkers and enabling them to receive brief intervention.
